# Supplementary material for: An Increase in HSF1 Expression Directs Human Mammary Epithelial Cells toward a Mesenchymal Phenotype
Source: Cancers (Basel). 2023 Oct 12;15(20):4965. doi: 10.3390/cancers15204965 (PMC10605143; doi:10.3390/cancers15204965)
Supplement: Supplementary file 1 [file cancers-15-04965-s001.zip › cancers-2641198-File S1.pdf]

An increase in HSF1 expression directs human mammary epi-thelial cells toward a mesenchymal phenotype.

Original Images for Blots

Fig.1a.

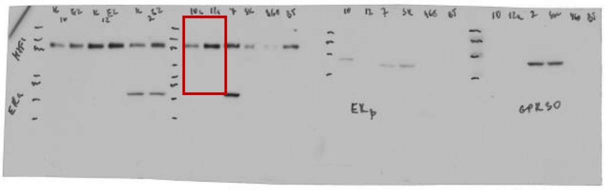

HSF1

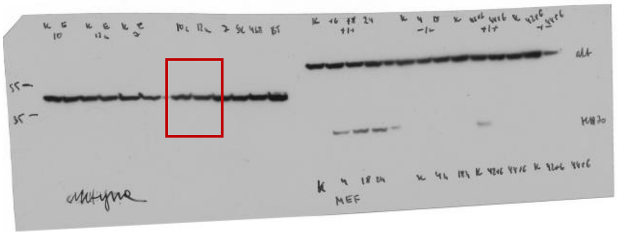

ACTB

Fig.1b.

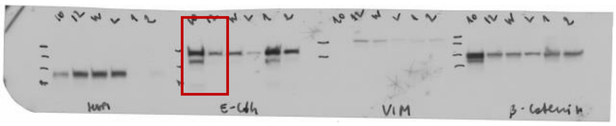

CDH1

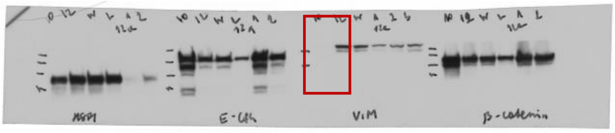

VIM

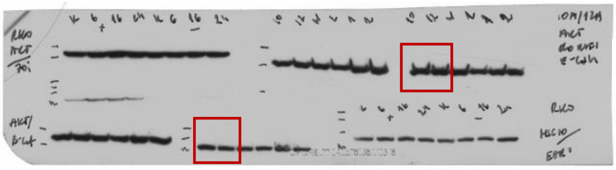

ACTB

HSPA8

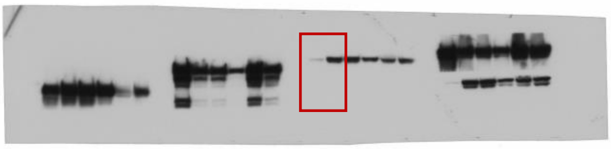

SNAI2

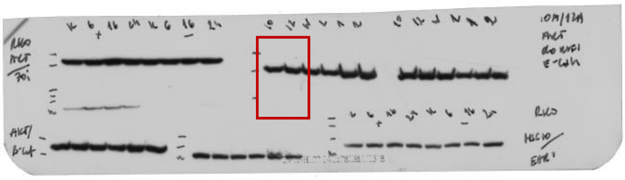

ACTB

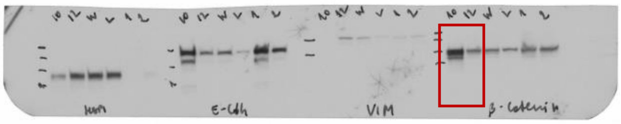

CTNNB1

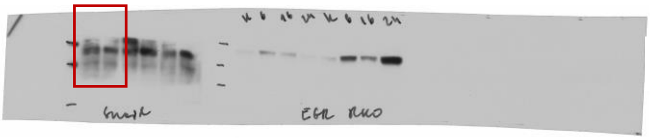

SNAI1

Fig.2a.

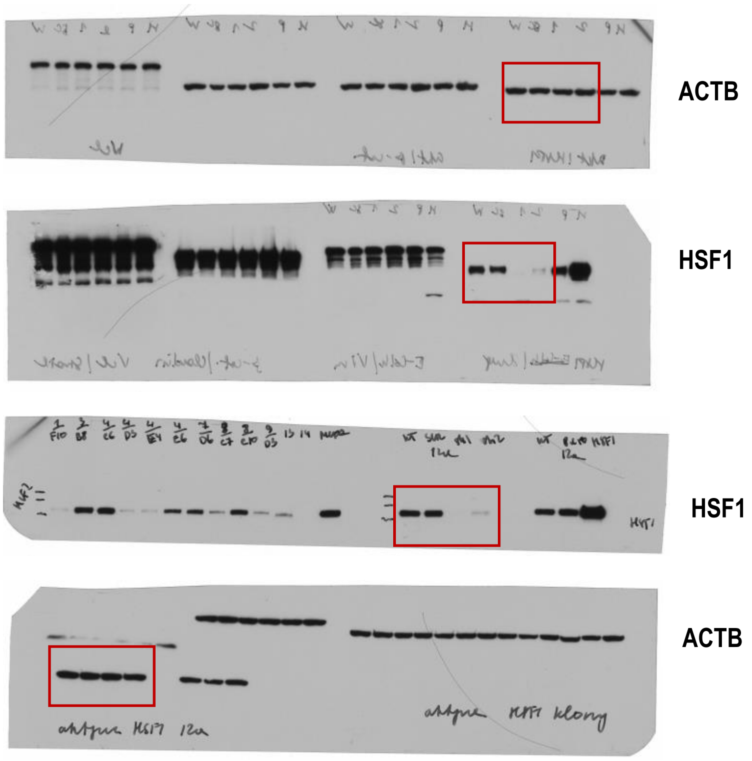

Fig.3a

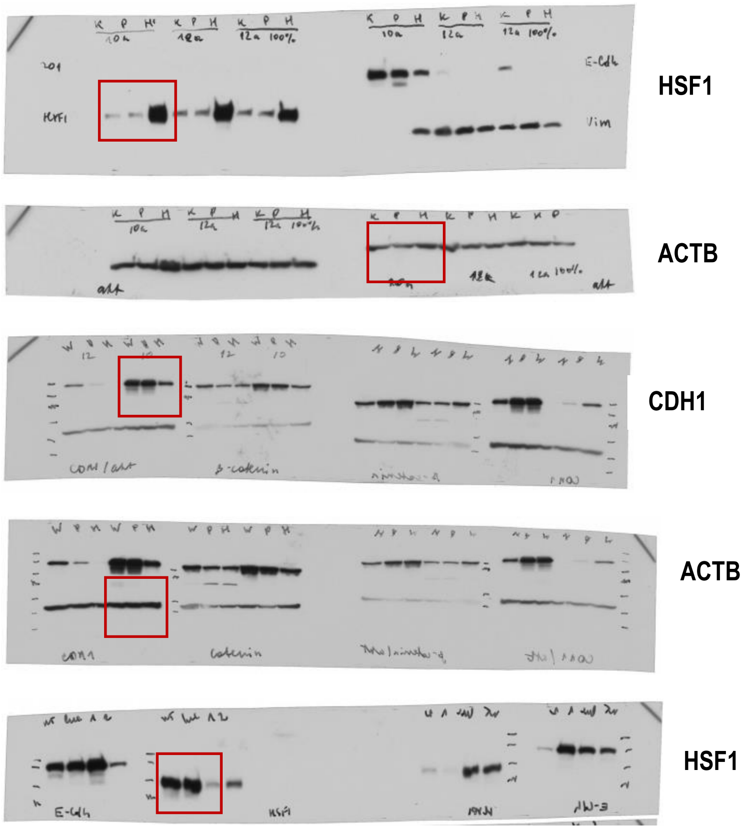

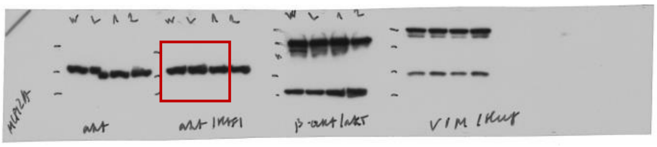
ACTB

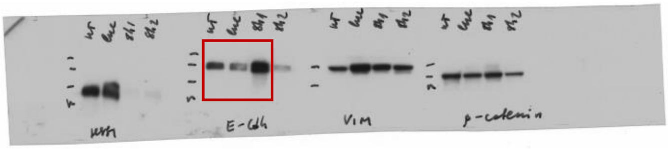
CDH1

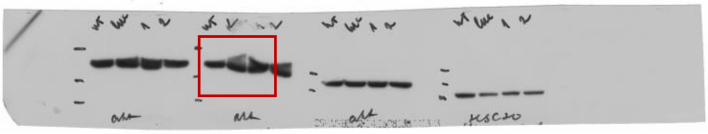
ACTB

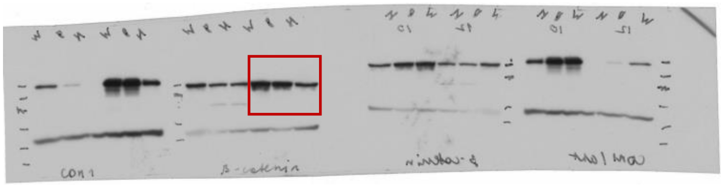
CTNNB1

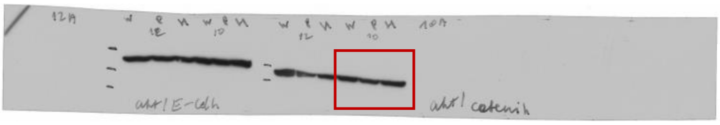
ACTB

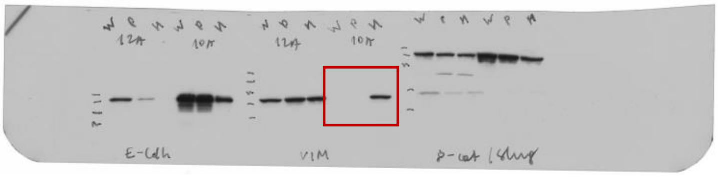
VIM

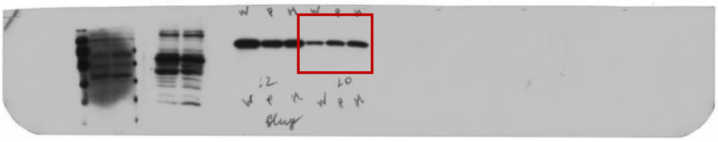
SNAI2

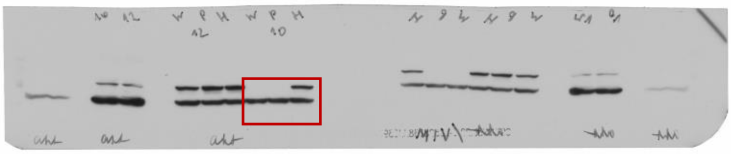
ACTB

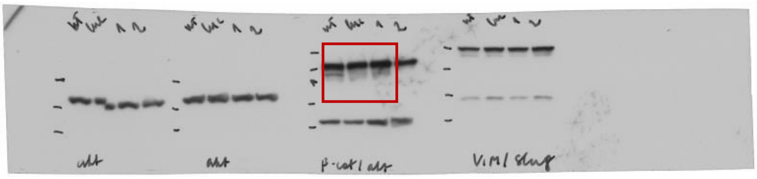
CTNNB1

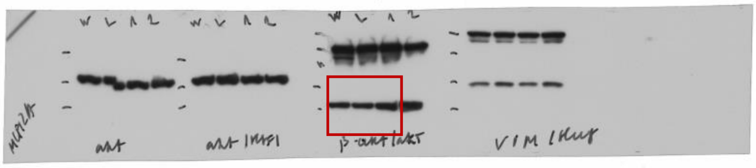
ACTB

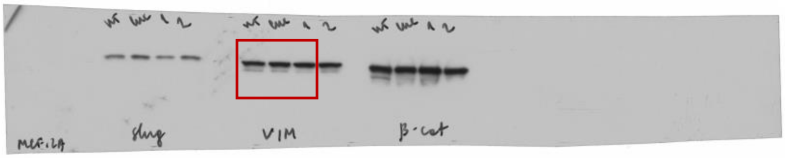

VIM

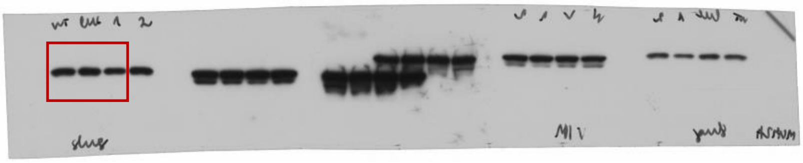

SNAI2

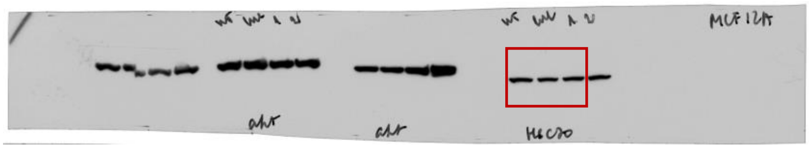

HSPA8

Fig.5a

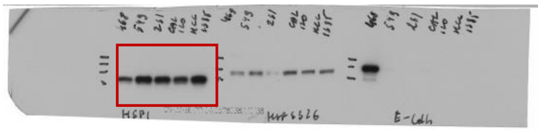

HSF1

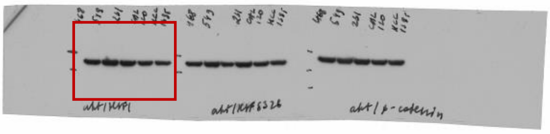

ACTB

Fig.5b

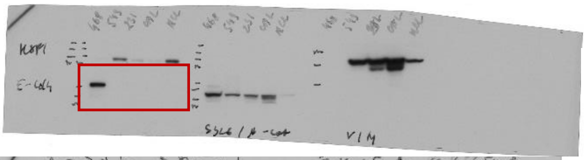

CDH1

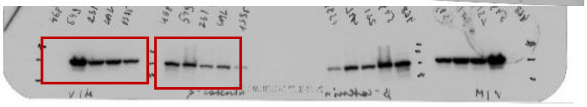

VIM CTNNB1

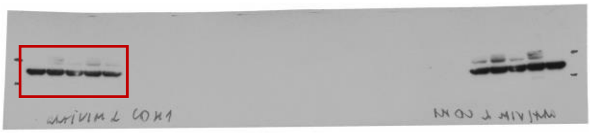

ACTB

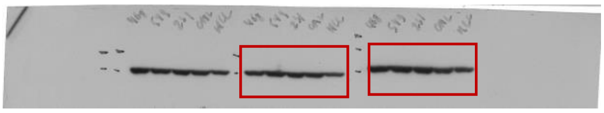

ACTB

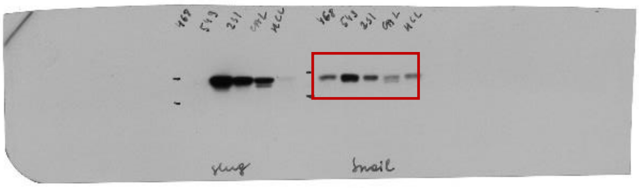

SNAI1

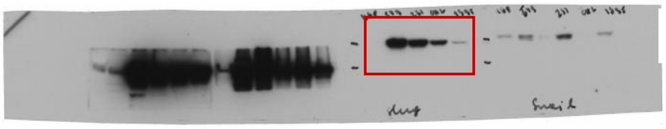

SNAI2

Fig.6a.

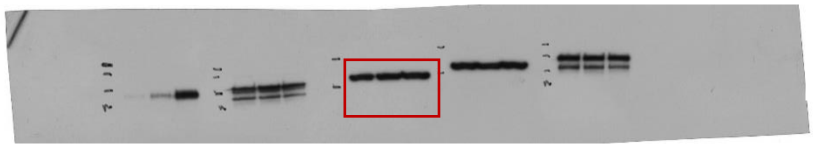

ACTB

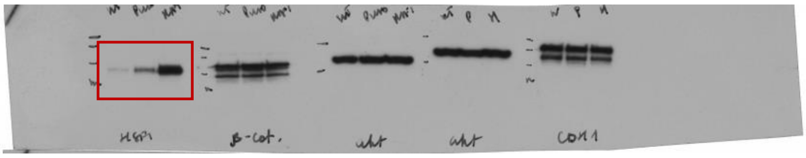

HSF1

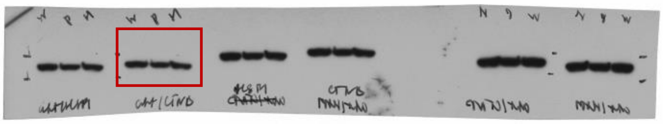

ACTB

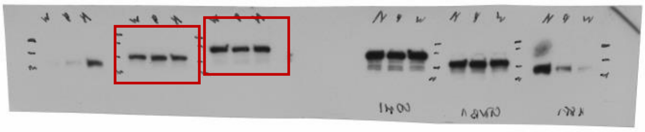

CDH1 CTNNB1

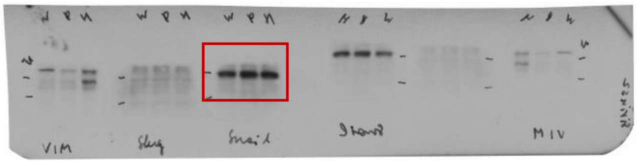

SNAI2

Fig.7b

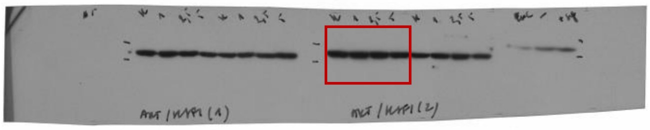

ACTB

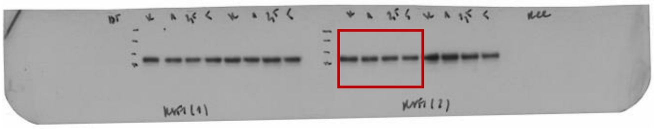

HSF1

Fig. 7c

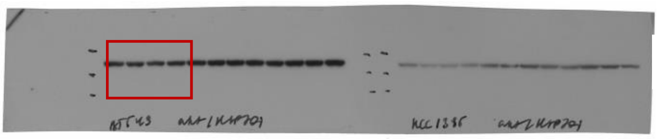

ACTB

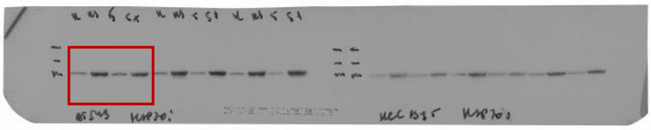

HSPA1A
